# Silicone Materials for Flexible Optoelectronic Devices

**DOI:** 10.3390/ma15248731

**Published:** 2022-12-07

**Authors:** Anna S. Miroshnichenko, Vladimir Neplokh, Ivan S. Mukhin, Regina M. Islamova

**Affiliations:** 1 Institute of Chemistry, Saint Petersburg State University, 7/9 Universitetskaya Emb., St. Petersburg 199034, Russia; 2 ChemBio Cluster, ITMO University, 49 Kronverksky Pr., St. Petersburg 197101, Russia; 3 Laboratory of Renewable Energy Sources, St. Petersburg Academic University, 8/3 Khlopina Str., St. Petersburg 194021, Russia; 4 High School of Engineering Physics, The Great St. Petersburg Polytechnical University, 29 Polytechnicheskaya Str., St. Petersburg 195251, Russia

**Keywords:** polysiloxanes, flexible optoelectronics, LEDs, III–V NWs

## Abstract

Polysiloxanes and materials based on them (silicone materials) are of great interest in optoelectronics due to their high flexibility, good film-forming ability, and optical transparency. According to the literature, polysiloxanes are suggested to be very promising in the field of optoelectronics and could be employed in the composition of liquid crystal devices, computer memory drives organic light emitting diodes (OLED), and organic photovoltaic devices, including dye synthesized solar cells (DSSC). Polysiloxanes are also a promising material for novel optoectronic devices, such as LEDs based on arrays of III–V nanowires (NWs). In this review, we analyze the currently existing types of silicone materials and their main properties, which are used in optoelectronic device development.

## 1. Introduction

Polysiloxanes are polymers containing alternating silicon and oxygen atoms in the main chain, with side organic substituents *R (*–*R*’*R*’’*SiO*–*)_n_* attached to each silicon atom, where *R* can be, for example, alkyl, aryl, vinyl, hydride, or other groups [1,2].

Polysiloxanes and materials based on them (silicone materials) have a number of useful properties, such as high thermal, frost stability (glass transition temperature is c.a—123 °C, crystallization temperature is c.a –90 °C and melting temperature c.a –50 °C) [3], ozone and chemical resistance, which have been applied as sealants, insulators, protective coatings, etc. Polysiloxanes are also employed in the food industry (kitchen utensils), medicine (implants), and “lab-on-a-chip” diagnostic devices, owing to their biocompatibility [4,5]. Polysiloxanes are of great interest in optoelectronics due to their high flexibility, good film-forming ability, and optical transparency.

A review paper in 2016 [6] described in detail the possibilities of using polysiloxanes in the composition of liquid crystal devices [7,8], computer memory drives [9], organic light emitting diodes (OLED) [10,11], and organic photovoltaic devices [12], including dye synthesized solar cells (DSSC) [6,13,14] and optical waveguides [15,16]. Polysiloxanes could be applied in the field of inkjet printing to create flexible transistors and batteries [17]. There have also been reports on polysiloxane-based materials possessing self-assembling behavior, which could be utilized in future for advanced optoelectronic devices [18,19].

The use of polysiloxanes in optoelectronics is not limited to the applications mentioned above. The synthesis of modified silicone (nano)composites and copolymers, with a wide range of functional side groups of the base chain synthesis, obtains silicone materials with novel properties such as, semiconductor behavior, photoluminescence, etc. [1,2,20,21].

Elastic optoelectronic and electronic devices have been suggested as an expansion of the well-established rigid semiconductor technology to the novel area of deformability, which is mainly required for biomedical, mountable, and wearable applications. Silicone materials are required for many optoelectronic devices, especially for flexible light-emitting diodes (LEDs). 

Nanocomposites based on silicone materials with various fillers, such as carbon nanotubes, graphene, and silver and copper nanowires (NWs), are used as flexible electrodes and wearable electronic devices [22,23,24,25,26,27,28,29,30]. Silicone nanocomposites with various luminescent fillers can be employed as dielectric light-emitting layers in flexible electroluminescent devices [31,32,33] and OLEDs [10,11]. Due to their good film-forming and elastic properties, silicone materials have been suggested as membranes for encapsulating arrays of III–V semiconductor nanowires (NWs). Flexible light-emitting devices with a relatively simple architecture could be based on silicone membranes with embedded NWs, where transparent electrodes are achieved by single-walled carbon nanotubes (SWCNTs) deposition on both sides of the membrane [34,35,36,37,38]. Furthermore, silicone materials can be used in metal-halide perovskite light emitting diodes (PLED) as protective layers in order to prevent perovskite oxidation in the environment [39].

The most commonly used semiconductor LED design is based on a p-n junction with a narrow bandgap active region between the p- and n-doped layers (emitters) [40,41,42]. However, the mechanical and geometrical properties limit their application for deformable optoelectronics, mainly due to the rigid substrates used for the epitaxial synthesis of the device structure. One of the most efficient methods by which to modify the thin film LED architecture for flexible applications is microLED formation and transferring to a flexible support [43]. In terms of mechanical properties, among the best materials for flexible LED devices are polyimide [44] and polydimethylsiloxane (PDMS) [34,36]. 

Therefore, polysiloxanes and silicone materials are very promising in the newer branches of optoelectronics. In this regard, the aim of this review is to analyze the currently existing types of silicone materials, mostly reported during the last 5−6 years, and their main properties in accordance with their utilization in optoelectronic device development (especially, for LEDs based on arrays of III−V NWs).

## 2. The Main Properties of Silicone Materials for Optoelectronics

### 2.1. Transparency, Morphology, and Adhesion

A typical representative of polysiloxanes is PDMS, which is optically transparent in the UV-visible and near-IR spectral ranges. PDMS has a low refractive index of 1.41 (at λ = 589 nm), and good film-forming properties and homogeneous morphology [45].

Commercially available PDMS Sylgard 184 forms transparent silicone rubbers (crosslinked polysiloxanes) at room temperature [46], so they are predominantly used in optoelectronic devices. First of all, Sylgard 184 is employed in flexible transparent electrodes for wearable electronics, biosensors, and ‘‘skin electronics’’ [25,28,29,30]. Moreover, Sylgard 184/Ag NWs composite has been applied in flexible piezoelectric element fabrication [47].

Elastic silicones have been rigorously studied as the mechanical basis for semiconductor LEDs since the publications of GaP-based thin film [48] and NWs-based [49] LEDs in a PDMS matrix in 2009 and 2010, respectively. Semiconductor NWs are a very promising alternative to the thin films due to their properties of high deformation sustainability, useful optical properties, such as waveguiding and light extraction along the wire axis [50], as well as adaptation for selective growth [51] and release from the growth substrate. An NWs-based LED can be considered an array of thin film microLEDs in terms of their functionality, but demonstrating significantly higher material quality and not requiring complex and expensive microstructuring post-growth processing. NW arrays can be synthesized on inexpensive lattice-mismatched substrates, e.g., silicon wafers or even metal foil or plastics; moreover, after the NW array release, the growth substrates can be mechanically and chemically treated and repeatedly used in the manner of a cooking dish [52,53,54].

The general concept of an NWs/PDMS membrane device is to transfer the vertical array of NWs into the silicone matrix (predominantly Sylgard 184), wherein the top and bottom NW parts are revealed for electrode deposition [34]. Generally, the NWs contain the active region, where the radiative recombination occurs, while the NW tips serve as the p- and n-type conductive materials (emitters) for carrier injection from the external electric circuit to the active region inside the NW. The intrinsic structure of NWs are normally organized in the axial geometry, when the active region represents a disk of the narrow bandgap material sandwiched between the p- and n-doped segments, or in the core-shell or radial geometry, when the active region is manifested by the ring cylinder of a narrow bandgap material between the conductive core and shell n-/p-type material. In the latter case, the shell material at the top of the NW part is connected to the electrode of one polarity, and the revealed core material in the bottom NW part is connected to the opposite polarity. NWs/PDMS membrane LEDs require at least one transparent electrode for the light extraction, while the other electrode can be intransparent. Therefore, one of the most efficient fabrication methods is based on the use of flexible copper tape, serving as a mechanical support for peeling-off and, simultaneously, as an electrode to the top parts of the NW array [34,55]. After the membrane is released from the substrate, the bottom NW parts are revealed, so the front contact of a transparent conductive material can be directly deposited.

Silicone material for flexible devices based on NWs/PDMS membranes should satisfy certain specific demands. First, the silicone material should be elastic and strong enough to provide sufficient mechanical support. The problem arises at the fabrication stage, since the most common method of NWs/PDMS membrane release is peeling, which is challenging for the PDMS matrix. Sparse arrays of long (i.e., more than 15 µm in length) NWs can be easily peeled from the epitaxial substrate, since the PDMS membrane is thick enough to sustain the deformation experienced during the peeling process. However, for dense arrays of short (less than 5 µm in length) NWs, the PDMS matrix is thin and fragile, so the strong force applied at peeling inevitably leads to the PDMS material destruction manifested by tears and multiple micrometer scale holes. It has been determined that the main reason for this membrane destruction is the high adhesion of the commonly used Sylgard 184 to the epitaxial substrate [38,56]. While for the sapphire wafers and sparse arrays of long InGaN/GaN NWs, this adhesion is negligible because, at peeling, the force application event rarely occurs and the stress effectively relaxes with a 15−20 µm thick membrane. 

**Figure 1:**
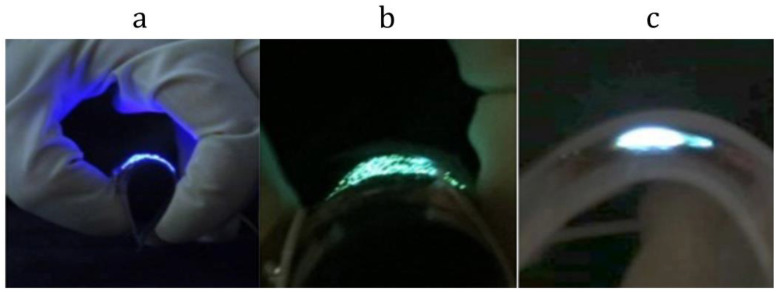
Optical images of operating flexible blue (**a**), green (**b**), and white (**c**) Sylgard/InGaN NW LEDs [34,40,58].

Flexible NW/PDMS LEDs have high stability of blue pixels since III−V, especially III−N semiconductors, demonstrate high stability in terms of material parameters even at high current densities. Compared to most commercially available OLEDs, NW/PDMS LEDs can also maintain their performance after bending and stretching cycles (up to 30 cycles) [10,57]. First, flexible green and blue light-emitting diodes based on arrays of InGaN NWs in a Sylgard 184 matrix [34,58] were demonstrated in 2015 by M. Tchernysheva’s group. Later, in 2016, white LEDs were reported in [35] (Figure 1). The blue flexible LED is presented in Figure 1a. InGaN NWs feature lower indium (In) content compared to the green LED shown in Figure 1b. The white LED presented in Figure 1c has yellow phosphorus particles in the PDMS membrane, providing the down conversion of NW emission light.

In 2021, a flexible and stretchable LED based on InGaN NWs/Sylgard 184 membranes with a flexible contact made of single-walled carbon nanotubes (SWCNTs) was demonstrated [36]. In the same year, the authors of [29] reported on a flexible red LED based on arrays of GaPAs/GaP NWs in a Sylgard 184 matrix [37].

Despite widespread use, Sylgard 184 has significant drawbacks such as high adhesion to silicon substrates [59]. Thus, it can be challenging for the exfoliation of array of 3–5 µm long GaP NWs grown on Si wafers and optoelectronic device fabrication [56]. These limitations can be overcome by the usage of PDMS copolymers (modified analogs), such as block–copolymers PDMS, polyester–*block*–polyurethane [60], and PDMS–*block*–polyester [61], which have transparency and flexibility comparable to PDMS, as well as other improved characteristics (decrease in adhesion, increase in tensile strength, etc.). It has been shown that PDMS can be modified by polystyrene side-chain grafting, so the developed PDMS-*graft*-polystyrene have significantly reduced adhesion to the Si, allowing efficient peeling of the record thin large area NWs/PDMS membranes without PDMS matrix damage [38,56].

**Figure 2:**
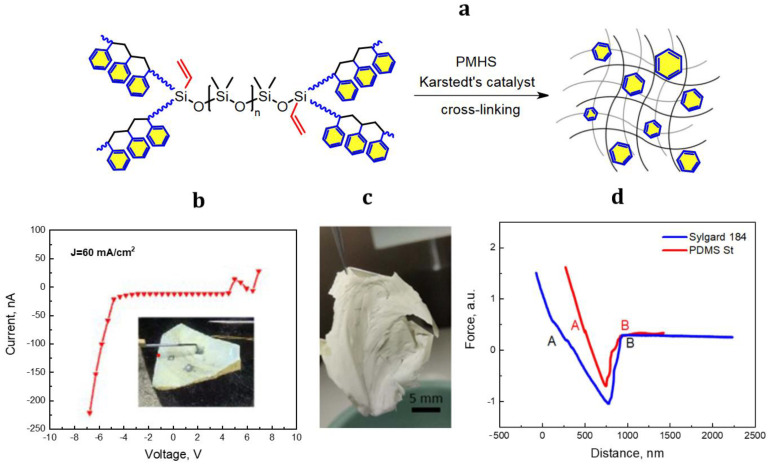
Scheme of PDMS-*graft*-polystyrene cross-linking (**a**), current-voltage characteristic (**b**). Reproduced with permission [38]. Copyright 2020, Royal Society of Chemistry. Optical photograph (**c**) of the released PDMS-*graft*-polystyrene (PDMS St)/n-GaP membrane, atomic force microscopy (AFM) approach/retraction curves showing the adhesion of silicone rubbers to Si substrate (distances between points A and B corresponds to the cantilever piezo displacement) (**d**) [56].

In 2020, a flexible Schottky barrier diode was presented [38,56], in which PDMS-*graft*-polystyrene was used as a flexible supporting substrate for n-GaP NW arrays. Polystyrene grafting to PDMS achieves an adhesion value to the growth silicon substrate of 0.66 from the value of Sylgard 184 (Figure 2). This provides an efficient transfer (releasing from the growth substrate) of n-GaP NWs arrays with a height of 6−8 µm over a relatively large area (3 square inch) [56].

Current-voltage characteristics of a p-doped SWCNTs/n-GaP NWs/ PDMS-*graft*-polystyrene membrane, shown in Figure 2b, demonstrates clear rectifying behavior, indicating no mechanical tears of the silicone matrix. Figure 2d presents the retract curves of a silicon cantilever to the standard PDMS and the modified PDMS-*graft*-polystyrene. It can be seen that the distances between points A and B are different, demonstrating lower adhesion of the PDMS-*graft*-polystyrene to Si.

**Figure 3:**
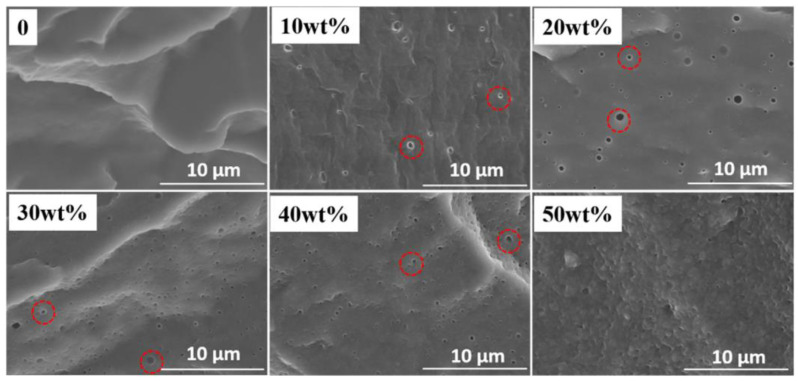
SEM images of the morphology of PDMS-*block*-PMMA blends at different loadings of PMMA (*wt* is weight percent). Red circles highlight supramolecular formations. Reproduced with permission [62]. Copyright 2021, Wiley-VCH.

However, PDMS-*graft*-polystyrene is opaque due to the formation of spherical supramolecular formations of side chains, which narrows the applicability of this copolymer in optoelectronics. The morphology of PDMS block-copolymers can also be inhomogeneous due to the formation of blends of one copolymer in another due to differences in the structure of the main chain and molecular weights. The latter case is typical for PDMS-*block*-PMMA copolymers, in which the blends size increases along with initial PMMA loading [62] (Figure 3).

**Figure 4:**
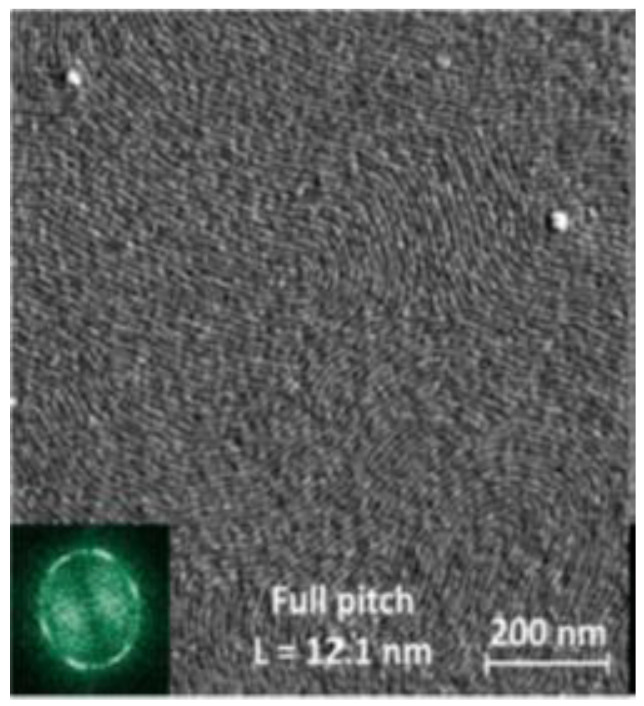
AFM phase images (1 μm^2^) of thermally annealed PDMS-*block*-PMMA thin film. Reproduced with permission [64]. Copyright 2015, American Chemical Society.

C.J. Hawker et al. demonstrated that the use of PDMS-*block*-PMMA helps to create domain periods as small as 12.1 nm, which is one of the smallest highly ordered nanoscale patterns (Figure 4). This opens up the possibility of creating nanoscale processors, transistors, and devices with high resolution by sub-10 nm lithography [63,64].

Optical transparency is desirable for polymer material in order for it to be used in optoelectronics. In this regard, a method for the preparation of transparent copolymers based on PDMS and PMMA was proposed in 2012 [65]. PMMA side chains were grafted to SigUMAx, which are PDMS macromonomers functionalized with 2-(methacryloxy)ethyl isocyanate (MOI). The transparency of the material was adjusted by changing the molar ratio MOI (x) in SigUMAx. Despite the improved optical properties, PDMS–*block*-PMMA copolymers have not been widely applied in the field of optoelectronics, because their preparation involves multiple stages of synthesis [62,63,65].

**Figure 5:**
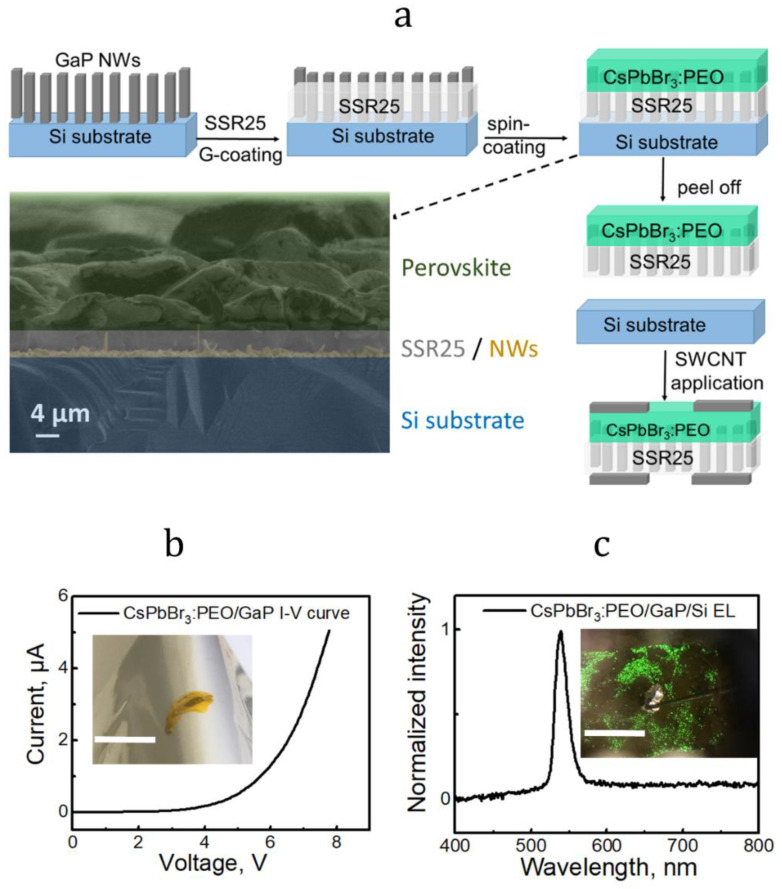
Scheme for creating an LED (**a**), I–V curve (**b**), and electroluminescence spectrum (**c**). Reproduced with permission [59]. Copyright 2021, American Chemical Society.

In 2021, it was demonstrated that introducing functional groups into the polysiloxane backbone obtained transparent silicone material with reduced adhesion to the silicon substrate. The introduction of phenylethylene substituents into the polysiloxane backbone reduces adhesion to the silicon substrate [59]. Thus, in [59], the preparation of transparent phenylethylene-functionalized silicone rubbers SSR25 was reported with a reduced adhesion value of 0.55 relative to Sylgard 184. The authors demonstrated the application of SSR25 as an encapsulating matrix for n-GaP NW arrays and presented a flexible green LED based on the integration of an SSR25/n-GaP NWs membrane and CsPbBr_3_ perovskite (Figure 5). 

The estimated open voltage for the reported PLED is about 4 V (Figure 5b), which is an adequate value for CsPbBr_3_ perovskite-based LEDs. PLED shows an electroluminescence (EL) line spectral position near 538 nm under an applied external voltage of 5 V (Figure 5c). The EL line corresponds to the optical transition in CsPbBr_3_. The inset in Figure 5b demonstrates the flexibility of the fabricated hybrid PLED membrane, while the optical image in the inset of Figure 5c shows low uniformity of luminescence, which can be associated with the non-uniformity of NWs/SSR25 distributed contact and layer of perovskite. Thus, the concept of NWs/PDMS membrane LEDs can be expanded for hybrid structures, where the array of NWs serves as the distributed electrode to the active region of a material different from the NWs and deposited onto the NWs/PDMS membrane surface. In this architecture, the NW/PDMS array provides both deformability and distributed electric contact to the active region made up of a variety of materials, which can be quantum dots, perovskites, organic, and any other electroluminescent materials. The main advantage of such a hybrid architecture is the exploitation of the high efficiency of the active region material, which is simply added to the ready-to-use NWs/PDMS membrane mechanical and electrical chassis, and does not require complex processing to package the active region inside the specific carrier transport layer. For example, metal halide perovskites CsPbX_3_ (X−Cl, Br, I) LEDs require specific electron and hole transport layer materials, such as ITO and C60 or F8, etc., which create difficulties for adaptation to deformable devices [66]. Meanwhile, the perovskite active layer in the planar geometry cannot exceed 200−300 nm because of low carrier diffusion length in the CsPbX_3_ material; therefore, the perovskite LED has a limited brightness. The NWs/PDMS membrane provides selective conductivity, which works in a similar way to electron/hole transport layers in the classic perovskite LED architecture, while the NW top parts can penetrate the perovskite active region material that improves carrier injection and allows for a higher perovskite layer thickness leading to the higher LED brightness [59,67]. 

The modified polysiloxanes can be used to improve the optical transparency and thermal stability of electrolytes for dye-sensitized solar cells (DSSC); for example, functionalized with imidazolium iodide [13] and cyclic sulfonium iodide [14] polysiloxanes. The use of poly[(3-N-methylimidazolium-propyl)methylsiloxane-*co*-dimethylsiloxane]iodides for highly efficient DSSC electrolytes was published in [68]. 

Thus, silicone materials and composites based on Sylgard 184 have been most widely used in optoelectronics. Due to their transparency, these materials can be applied in OLEDs, LEDs based on III–V NW arrays, solar cells, flexible piezoelectric elements, and body-worn electronics. In addition to Sylgard 184, alternative functionalized polysiloxanes and silicone materials based on them have been used in optoelectronics. 

### 2.2. Mechanical Properties and Self-Healing

Relatively high elasticity and tensile strength, along with transparency, are crucial for polymer materials applied in optoelectronic devices [59]. Cross-linked Sylgard 184 is characterized by a high (for applications in optoelectronics) elongation at break (*ε* = 100%), tensile strength (*σ* = 2.4 MPa), and Young’s modulus (*E* = 1.1 MPa) [21]. Other silicone materials based on modified polysiloxanes have also found application in optoelectronics, the main tensile characteristics of which are presented in Table 1.

Obtaining graft- and block-copolymers enables the improvement of mechanical properties of PDMS (Table 1). For example, PDMS-*graft*-polystyrene, which is slightly inferior to Sylgard 184 in terms of *σ*, has a large Young’s modulus, *E* = 1.9 MPa. Higher tensile strength provides easier membrane release from the growth substrate without the risk of mechanical damage. Thus, PDMS-*graft*-polystyrene has been used to create flexible optoelectronic membranes with a large area of 3 square inches [56]. In addition, PDMS–*blok*–PMMA copolymers can be used in silicon wafer patterning [63,64]. Tensile properties of PDMS-*block*-PMMA copolymers could be controlled via PMMA loading and can reach almost twice as high a tensile strength as Sylgard 184 (Table 1). PDMS-*block*-polyurethanes demonstrate high values of σ for polysiloxanes at a relatively high elasticity ε (Table 1). In 2022, electrically conductive elastic ferrocenyl-containing rubbers (EFSR) obtained by catalytic hydrosilylation crosslinking between ferrocenyl-containing silicone rubber and vinyl-containing PDMS were presented [69]. EFSR shows almost 1.5 times higher tensile strength and almost twice higher elasticity compared to Sylgard 184 (Table 1). The polyester-*block*-PDMS copolymers with extremely high values of *ε* = 778–815% compared to the abovementioned PDMS block-copolymers were reported in [60].

Polysiloxanes possess self-healing properties—the ability to partially or completely restore their original characteristics after mechanical damage; for example, cracks and ruptures [77].

These self-healing properties open up new possibilities for creating flexible self-healing protective coatings, screens, electrodes, and solar cells. Self-healing silicone materials can be obtained on the basis of covalent interactions, including reversible Diels-Alder [78,79,80,81,82,83], ester [84], imine [85], and disulfide bonds [86,87].

In 2016, the preparation of a self-healing polymer-metal complex (PMC) 2,2’-bipyridine-5,5’-dicarboxyamide-*co*-PDMS with Fe^2+^ and Zn^2+^ ions as a metal center and counterions Cl^−^, BF_4_^−^, ClO_4_^−^, and CF_3_SO_3_^−^ was described. Zn–PDMS and Fe–PDMS possess electrical conductivity at the antistatic level and, according to [70], can be used in organic field-effect transistors (OFETs) as gate dielectrics and in wearable electronics. The self-healing efficiency of Zn(CF_3_SO_3_)_2_–PDMS reaches 76% at room temperature after 48 h.

In 2017, Yu D. et al. [71] demonstrated a self-healing PMC with Cu^2+^ as a metal center (Cu–PDMS), achieving a self-healing efficiency of 87% at 30 °C for 1 h. In 2021, self-healing PMCs based on pyridine-2,6-dicarboxamide-*co*-polydimethylsiloxanes (Py–PDMS) with Co^2+^ and Ni^2+^ metal centers were presented [72,73]. The self-healing efficiency for Ni–Py–PDMS25000 and Co–Py–PDMS25000 reached 92% and 96%, respectively, at room temperature. Ni–Py–PDMS25000 and Co–Py–PDMS25000 have sufficient elongation at a break of about 2100% and 1800%, respectively. It has been shown that the tensile and self-healing properties of PMCs can be controlled by changing the metal/ligand ratio, the average molecular weight of ligands and the loading metal. In 2021, self-healing materials based on Py–PDMS PMCs were further developed and Tb- and Eu-containing PMCs (Tb–Py–PDMS and Eu–Py–PDMS, respectively) were presented [20]. It has been demonstrated that the incorporation of Eu^3+^ and Tb^3+^ as metal centers enables sufficient elongation at break of 450%, and a self-healing efficiency of 80% for 36 h at room temperature, along with a strong photoluminescence (PL) response. In 2022, it was demonstrated that the replacement of a tridentate ligand with a tetradentate 6,6’-bipyridine-carboxamide leads to the formation of coordinatively saturated Ln-Bipy-PDMS PMCs and an increase in tensile strength up to *σ* = 1.5 MPa compared to Ln–Py–PDMS (*σ* = 0.45 MPa). The mechanism of self-healing in this case becomes non-autonomous; the efficiency of self-healing was 90% within 48 h at 100 °C [74]. Self-healing luminescent PMCs could be applied as flexible photoluminophors for optoelectronic devices (detailed in chapter 1.3. Luminescent properties). Terpyridine lanthanide-incorporating polysiloxanes with Mn = 25000 of PDMS units (H–PU–Ln), presented in [75], showed similar Py-PDMS elongation at a break of 498% and a tensile strength *σ* = 0.58 MPa. The Young’s modulus for H– PU– Ln was *E* = 1.7 MPa. Terpyridine lanthanide-incorporating polysiloxanes with Mn = 5000 of PDMS units (L–PU–Ln) demonstrated slightly lower tensile strength *σ* = 1.2 MPa than Ln–Bipy–PDMS PMCs. However, L–PU–Ln have an extremely high value of Young’s modulus *E* = 13.2 MPa, along with low elongation at break *ε* = 20%. H–Pu–Ln reaches a self-healing efficiency of 27.5% after 48 h at room temperature. Analogously to Ln–Py–PDMS and Ln–Bipy–PDMS, we would further call H–PU–Ln and L–PU–Ln as Ln–Tpy–PDMS25000 and Ln–Tpy–PDMS5000, respectively.

In 2022, Miao Tang et al. presented flexible self-healing silicone materials based on PDMS-*block*-dithiothreitol copolymers with a tensile strength of *σ* = 0.43 MPa and an elasticity of *ε* = 1500% [76]. The self-healing efficiency of these materials reaches 100% at room temperature in just 30 s after damage, which is one of the fastest self-healing elastomers available today.

Thus, tensile properties such as high elasticity and sufficient mechanical strength are crucial for polymers applied in optoelectronics. Widely used in various optoelectronic devices, Sylgard 184 has relatively high *ε* and σ values, which can be improved by using, for example, PDMS copolymers. However, in optoelectronics, it is required to have a set of properties, namely, improved mechanical characteristics, along with the optical transparency of the material. Integrated self-healing silicone materials, which are able to restore their integrity and self-repair mechanical damage, pave the way for future optoelectronic devices.

### 2.3. Luminescent Properties

**Figure 6:**
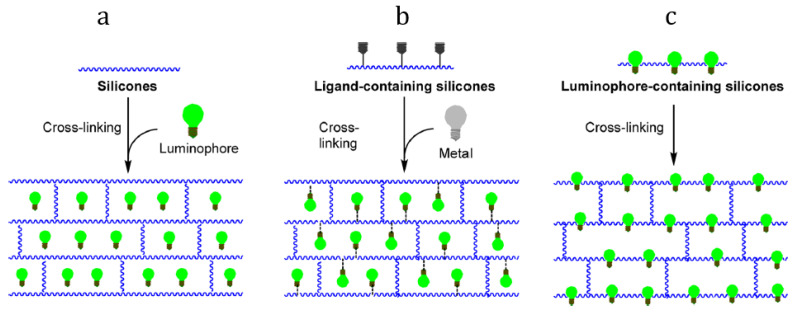
General approaches to luminescent silicone rubbers: use of fillers (**a**), metal binding (**b**), and copolymers (**c**). Reproduced with permission [88]. Copyright 2021, American Chemical Society.

Luminescent silicone materials are of great interest in optoelectronics as luminophores for flexible displays and devices. There are two fundamentally different ways to obtain luminescent silicone materials: (i) introducing various phosphors as fillers into liquid silicone compositions (for example, Sylgard 184) (Figure 6a) and obtaining solid luminescent composites (cross-linked silicone rubber); (ii) chemical modification of polysiloxane chains, and obtaining luminescent copolysiloxanes and materials based on them (Figure 6b,c) [88].

The introduction of organic and inorganic luminescent fillers is the simplest approach to obtain luminescent silicone materials. Inorganic luminescent fillers include quantum dots of doped graphene [89,90] and typical semiconductors CdSe, CdS, and ZnS, [31,32,91], as well as lanthanide-containing metal-organic frameworks [92].

**Figure 7:**
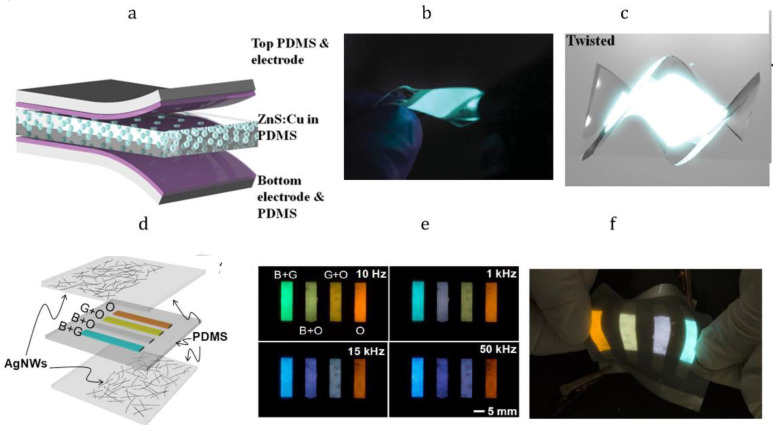
Blue ACELs architecture (**a**), optical image of blue twisted ACEL with (**b**) and without (**c**) applied voltage. Reproduced with permission [31]. Copyright 2015, Wiley-VCH. Colorful ACEL architecture (**d**) optical image of colorful ACEL under applied voltage (**e**) and stretched (**f**). Reproduced with permission [33] Copyright 2018, Nature Publishing Group.

ZnS quantum dots as fillers for PDMS provide electroluminescent of silicone materials under an applied high voltage of up to 200 V. With the use of this approach, a stretchable blue alternating current electroluminescent device (ACEL) was fabricated [31]. Doping ZnS with copper (ZnS:Cu) and manganese (ZnS:Cu, Mn) atoms provides green and orange colors of electroluminescence, respectively. Intermediate yellow and violet colors can be achieved by the combination of the different quantum dots, which has been shown in stretchable ACELs (Figure 7) [31,32,33]. Moreover, the electroluminescence intensity of ZnS/PDMS remains the same, even after device stretching up to 100%.

Mechanochromic photoluminescent silicone materials with 1,1,2,3,4,5-pentaphenyl-1-hydro-silol and other organic phosphors were reported in [93]. Coordination compounds of rare earth metals can also act as luminescent fillers [94,95,96,97]. Therefore, silicone materials with a PL quantum efficiency of 33% were obtained by [Eu(tta)_3_(H_2_O)_2_] incorporation during the cross-linking via hydrosilylation reaction between tetramethyl-tetravinyl-cyclotetrasiloxane (D4Vi) and tetramethyl-tetrahydro-cyclotetrasiloxane (D4H) [95].

Another approach for luminescent silicone materials is the introduction of dual-function luminescent complexes into a polysiloxane matrix, which can simultaneously act as a catalyst and luminophore for crosslinking by the hydrosilylation reaction, [88,98]. In 2021, 2-phenylpyridine-triphenylphosphine-platinum chloride [Pt(ppy)Cl(PPh_3_)] was used as a luminescent filler and catalyst for the crosslinking of vinyl-terminated polydimethylsiloxane and polymethylhydrosiloxane [88]. The quantum yield of PL for the obtained silicone material was 12.5%.

**Figure 8:**
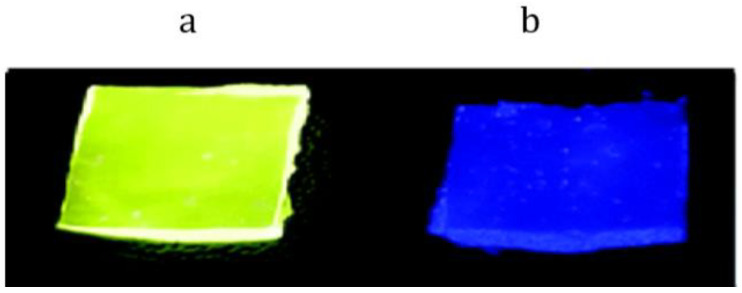
Optical photographs of thermo-photoluminescent silicone rubbers at 80 °C (**a**) and 120 °C (**b**). Reproduced with permission [98]. Copyright 2021, Royal Society of Chemistry.

Silicone rubbers were obtained via cross-linking by the hydrosilylation reaction of α,ω-polydimethylsiloxane and poly(dimethylsiloxane-*co*-methylhydrosiloxane), as published in [98]. The C,N-chelate deprotonated platinum(II) diaminocarbene complex(cis-[PtCl_2_(CNXyl)_2_]) was used as a catalyst. Silicone rubbers with cis-[PtCl_2_(CNXyl)_2_] exhibited temperature-dependent luminescence. The PL emission of these silicone materials irreversibly changes when heated from 80–100 °C (green radiation) to 120 °C or higher (blue radiation) (Figure 8).

**Figure 9:**
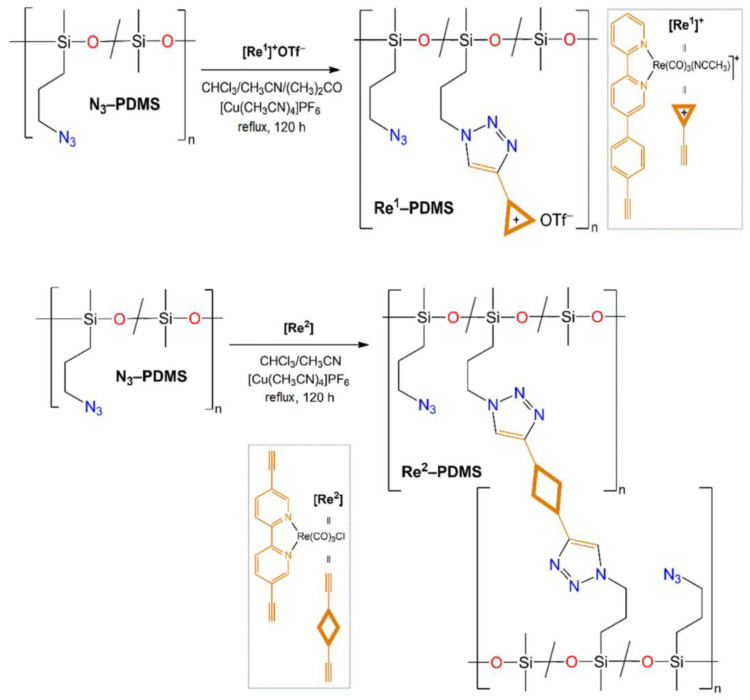
Scheme of Re^1^-PDMS and Re^2^-PDMS synthesis [99].

Despite the simplicity of the proposed approach, luminescent fillers can be washed out of the silicone material upon prolonged treatment with organic solvents [88]. Another approach to obtain luminescent silicone materials is the polysiloxane chain modification by various chemical reactions, including click chemistry. This approach provides a uniform distribution of phosphors in the polymer matrix and excludes their washing out by organic solvents. The preparation of silicone luminescent materials based on rhenium-containing polysiloxanes (Figure 9) PMCs by the reaction of azide-alkyne cycloaddition (CuAAC) in the presence of copper (I) catalyzed was described in [99]. However, the rhenium-containing PMCs (Re-PDMS) [Re(CO)_3_(MeCN)(5-(4-ethylphenyl)-2,2′-bipyridine)]OTf (Re^1^) and Re(CO)_3_Cl(5,5’diethynyl-2,2’-bipyridine)] (Re^2^) have shown low quantum yields (0.5%).

Lanthanide coordination compounds can be used to obtain silicone materials with a higher photoluminescence quantum yield. Due to the forbidden nature of the characteristic 4f–4f electronic transitions, lanthanide ions have a long photoluminescence lifetime and sharp spectral lines [100,101]. In order to achieve high PL quantum efficiency, a sensitizer ligand is required for the so-called “antenna effect”, such as pyridine (Py) [20], bipyridine [102], or other aromatic groups [101].

**Figure 10:**
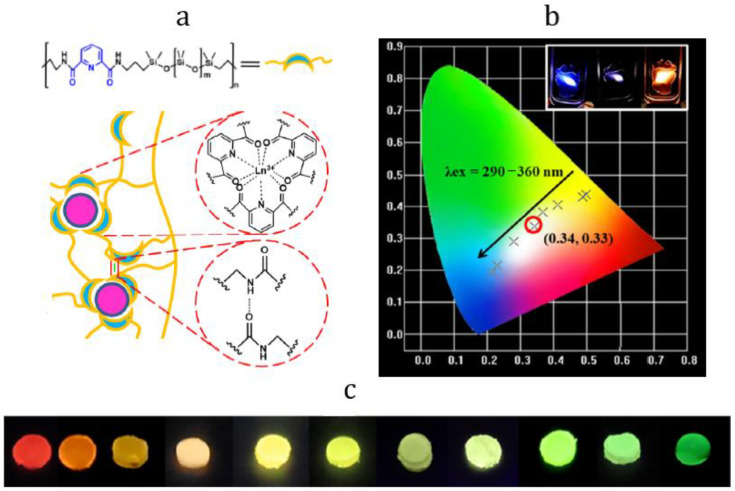
Schematic depiction of Ln−Py−PDMS and molecular structure of Py−PDMS (**a**), CIE 1931 color space (**b**), and optical photographs of Tb−Py−PDMS- and Eu−Py−PDMS-based phosphors (**c**) [20].

In 2020, H. Li et al. reported the preparation of photoluminescent self-healing terbium- and europium-containing PMCs (Tb−Py−PDMS and Eu−Py−PDMS) based on pyridinecarboxamide-*co*-polydimethylsiloxane [20]. The quantum yields for these materials were 40% and 30%, respectively. It was demonstrated that the photoluminescence color of PDMS can be controlled by Eu^3+^:Tb^3+^ ratio variating due to the sharpness of the lanthanides spectral line. By this approach, the authors managed to prepare a silicone material with emission color close to white light, which can be applied as a phosphor for white LEDs (Figure 10).

**Figure 11:**
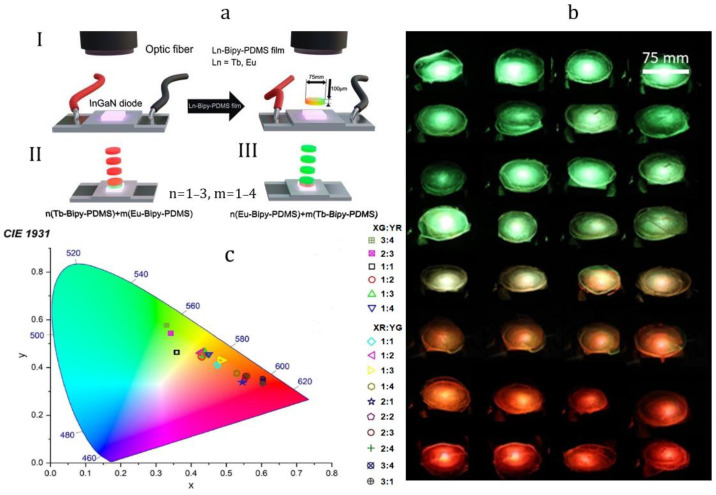
PL color tuning work-flow (**a**). Set-up scheme (I), first (II), and second (III) routes of the PL color tuning study. n and m reflect the numbers of red and green Ln-Bipy-PDMS films, respectively. Optical photographs stacked Tb−Bipy−PDMS and Eu−Bipy−PDMS films (**b**), and CIE 1931 color space (**c**). The notes “XG:YR”correspond to the stacks made of X green and Y red films, and “XR:YG” correspond to the stacks made of X red and Y green films. Reproduced with permission [74]. Copyright 2022, American Chemical Society.

In 2022 [74], terbium- and europium-containing PMCs with the tetradentate bipyridine ligand Eu−Bipy−PDMS and Tb−Bipy−PDMS were obtained. The photoluminescence quantum efficiency of Eu−Bipy−PDMS and Tb−Bipy−PDMS was 10.5 and 18.5%, respectively. Most importantly, photoluminescence color tuning can be achieved by simple stacking of 100 µm thick films. Due to non-autonomous self-healing, monolithic “sandwiches” were obtained from red and green films superimposed on each other via backing at 100 °C. Such monolithic “sandwiches” have two colors of photoluminescence, determined by the ratio and order of the films (Figure 11). Such monolithic “sandwiches” have two colors of photoluminescence, determined by the ratio and order of the films. The first film to the UV source mainly determines the color of emission due to an efficient absorption of the emitted light. Eu−Bipy−PDMS and Tb−Bipy−PDMS can be applied for fluorescent mosaics [103], lighting design, and optoelectronics as flexible self-healing screens [20,74].

**Figure 12:**
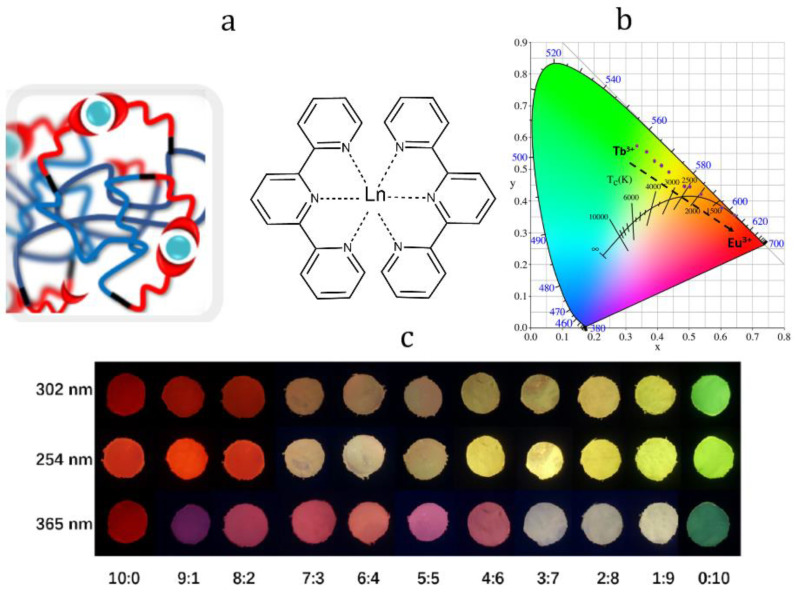
The proposed structure of Ln−Tpy−PDMS5000 (**a**), CIE 1931 color space (**b**), and optical images of Ln-Tpy-PDMS5000 (Ln: Eu and/or Tb) excited under 302, 254 and 365 nm (from right to left: Eu:Tb = 10:0, 9:1, 8:2, 7:3, 6:4, 5:5, 4:6, 3:7, 2:8, 1:9, 0:10) (**c**). Reproduced with permission [75]. Copyright 2022, Elsevier.

In [75], photoluminescence color tuning was shown for lanthanide-incorporating polysiloxanes with terpyridine ligand Ln−Tpy−PDMS5000. Quantum yield of both terbium- and europium-containing PMCs was around 28%. The PL color of Ln-Tpy-PDMS5000 can not only be tuned by Eu^3+^:Tb^3+^ ratio variation, but also by choosing the excitation wavelength (Figure 12). This is due to the Eu−Tpy−PDMS5000 excitation spectrum shape, which consists of a broadband absorption peak ranging from 250 to 400 nm and centered at 331 nm from the terpyridine ligands, together with a weaker and sharp peak at 395 nm from the Eu^3+^ intra-4f6 transition.

Therefore, luminescent silicone materials are mainly fabricated by the incorporation of luminescent fillers into the silicone composition or photoactive centers into the backbone of the polysiloxane. Luminescent silicone materials with semiconductor quantum dots [31,32,91] and graphene [89,90] fillers are used as light emitting layers in ACELs [31,32]. Silicone materials with organic complex fillers are employed as light emitting layers in OLEDs [57]. Chemical modification of the polysiloxane chain provides uniform distribution of phosphors in the polymer matrix and excludes the color center washing out by organic solvents. Using this approach, self-healing lanthanide-containing copolysiloxanes Ln−Py−PDMS and Ln−Bipy−PDMS with high quantum yields and long decay time have been obtained [20,74]. Such materials can be used as light-emitting layers for flexible displays, as well as self-healing protective coatings for smartphones, laptops, and tablet screens.

### 2.4. Electrical Conductive Properties

One of the most acute problems in deformable optoelectronic devices is the material for elastic highly conductive electrodes. The simplest and most affordable way to obtain electrically conductive polysiloxanes and materials based on them is the use of electrically conductive fillers (similar to the case of luminescent silicone materials). PDMS/CNT composites are well known due to their application as flexible and stretchable electrodes for optoelectronics, electronic skin devices, and wearable flexible devices, as well as neuronal implants [23,69,104]. PDMS/ Cu NWs have been suggested as flexible electrodes [30].

**Figure 13:**
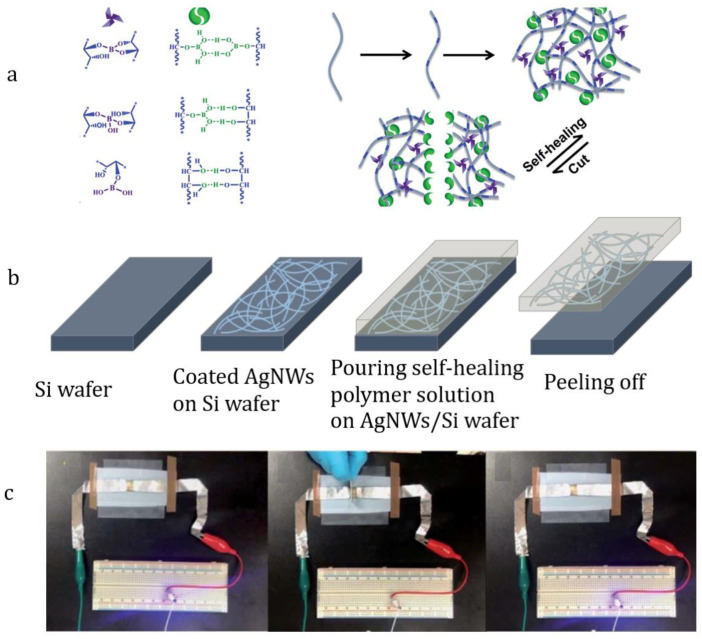
Scheme of PDMS-*block*-dithiothreitol preparation (**a**), flexible self-healing Ag NWs/PDMS-*block*-dithiothreitol electrode fabrication (**b**), and optical image of working electrode (**c**). Reproduced with permission [76]. Copyright 2022, Royal Society of Chemistry.

Silver nanowires (Ag NWs) are also commonly used as fillers [28,105]. In 2014, a flexible piezoelectric element based on Sylgard 184/Ag NWs was presented [47]. A AgNWs/PDMS hybrid electrode with a transmittance of 83% has a sheet resistance of 20 Ω/sq [105]. The Sylgard 184/Ag NWs composites have been employed as transparent electrodes for stretchable ACELs [31,33] and InGaN/PDMS LEDs [34,35]. Flexible self-healing electrodes based on Ag NWs/PDMS-*block*-dithiothreitol composites have been used in flexible optoelectronics and “electronic skin” (Figure 13) [76].

An electrically conductive PEDOT:PSS-*graft*-PDMS copolymer obtained by the PDMS surface functionalization with conductive poly(3,4-ethylenedioxythiophene)-styrenesulfonate PEDOT:PSS was introduced in 2019 [106]. The measured sheet resistance of the flexible tensile electrode based on PEDOT:PSS-*graft*-PDMS was 90 Ω/sq. At the same time, the change in resistance during deformation up to 100% was insignificant and did not change during 10000 cyclic stretching.

As the backside electrodes of LEDs can be intransparent, it has been proposed to use ferrocenyl-containing polymethylhydrosiloxane (FPS) as the electrode material for the NW top parts [38]. FPS features high elasticity and some electrical conductivity, so an electrode can be formed to the NW/PDMS membrane prior to the peeling. The FPS contact also provides additional mechanical support to the NW/PDMS membrane facilitating the peeling from the substrate. The best results were achieved with FPS mixed with multi-walled carbon nanotubes (MWCNTs) as a filler. The FPS/MWCNT material demonstrated high conductivity for the price of full intransparency [38]; therefore, it is suitable for the LED backside electrode.

In 2022, an elastic ferrocenil contaning silicone rubber EFSR was reported [69]. EFSR had a specific electrical conductivity of 9.5∙10^−12^ S/cm at an applied electric current of 1 Hz frequency, which corresponds to the electrical conductivity of antistatic materials [70,71,72]. The authors claimed that ferrocenyl units helped to achieve a high charge injection capacity without changing the microelectrode (EFSR) area in the neuronal implant, and consequently prevented the neuronal tissue damage during electrical stimulation. The incorporation of 5% wt carbon nanotubes into EFSR provided a specific electrical conductivity of 7∙10^−5^ S/cm. Such electrical conductivity values are comparable to the electrical conductivity of semiconductors; therefore, EFSR–CNT composites have been used as neuroimplants [69].

Therefore, there are two main approaches to obtain electrically conductive polysiloxanes: the introduction of conductive fillers into PDMS and modification of polysiloxane chains with electroactive centers. PDMS composites with graphene, CNTs, Ag NWs, and Cu NWs have become widespread as flexible and stretchable electrodes for optoelectronic and skin electronics devices [23,24,25,30,104]. In addition to composite silicone materials, there have been electrically conductive ferrocenyl-containing polysiloxanes and materials based on them, which have been used as flexible electrodes for Schottky diodes [69] and as neuronal implants [75]. Nevertheless, the potential of using modified conductive polysiloxanes is far from having been fully explored. 

## 3. Conclusions

The usage of polysiloxanes and silicone materials based on them are increasing, along with the development of new fields in optoelectronics (especially, for NWs-based LEDs).

Optical transparency and morphology homogeneity contribute to the widespread use of PDMS and silicone materials based on them in optoelectronics. Thus, silicone materials based on the commercially available silicone composition Sylgard 184 are employed as a matrix for organic emitters in OLEDs, and a flexible support matrix for flexible LEDs based on III−V NWs arrays [67,76,77,78,79]. In addition to Sylgard 184, functionalized polysiloxanes and materials based on them, which have a reduced adhesion to a growth silicon substrate, have been used in flexible III−V optoelectronics. Examples of this are phenylethylene-functionalized silicone rubbers [11] and PDMS-*graft*-polystyrene copolymers [69]. PDMS-*block*-PMMA and PDMS-*block*-polystyrene copolymers are used to pattern silicon wafers and further create sub-nanometer transistors, capacitors, and processors.

Self-healing luminescent silicone materials that can be used as flexible photoluminophores are very promising for the development of flexible optoelectronics. In the future, this paves the way for creating flexible self-healing displays, solar panels, piezoelectric generators, and capacitors.

Composites of PDMS with graphene, CNTs, Ag NWs, and Cu NWs have become widespread as flexible tensile electrodes for optoelectronic and skin electronics devices [61,62,63,67,73,74]. The extensible self-healing electrode Ag NWs/PDMS-*block*-dithiothreitol has also been suggested. In addition to PDMS-based composites, electrically conductive ferrocenyl-containing polysiloxanes nanocomposites, which have been used as flexible electrodes for Schottky diodes [38] and as neuronal implants [69], have also been proposed.

Luminescent silicone materials with semiconductor quantum dots [31,32,91], graphene [89,90], or organic complexes [25,26,50,86] are used as light emitting layers in ACELs and OLEDs [10,31,32,33]. Lanthanide-containing self-healing copolysiloxanes Ln-Py−PDMS, Ln−Bipy−PDMS, and Ln−Tpy−PDMS [20,74,75], which have high quantum yields and long decay, can be an alternative to composite silicone materials. Ln−Py−PDMS, Ln−Bipy−PDMS, and Ln−Tpy−PDMS can be used as light-emitting layers for flexible displays, as well as self-healing protective coatings.

Thus, silicone materials are being widely used in various fields of optoelectronics, the demand for which is growing every year. Flexibility, optical transparency, and biocompatibility of polysiloxanes make them promising materials for flexible optoelectronic devices. Polysiloxanes can be easily modified via chemical reactions or the introduction of different fillers, which obtain silicone materials with useful properties such as improved adhesion, semiconductor behavior, and photoluminescence, etc. However, most functional polysiloxanes are only synthesized in laboratory conditions and in relatively small amounts. We consider the main obstacle for their practical application is the development of a scaling-up process for functional polysiloxanes and silicone materials manufacturing.

## Figures and Tables

**Table 1:** Tensile properties of silicone materials used in optoelectronics.

*Silicone Material*	*σ*, *MPa*	*ε, %*	*E, MPa*	*References*
Sylgard 184	2.4	92	1.1	[46]
EFSR	3.5	170	1.4	[69]
PDMS-*graft*-polystyrene	1.5	90	1.9	[59]
PDMS–*block*–PMMA (wt* = 56%)	4.7	61	–	[62]
PDMS–*block*–PMMA (*wt* = 6%)	1.3	158	–	[62]
PDMS, polyester–*block*–polyurethane	14.3	92	–	[61]
Polyester-*block-*PDMS	0.5	815		[60]
Zn(CF_3_SO_3_)_2_–PDMS	0.6	310	1.1	[70]
Cu–PDMS	0.39	171	–	[71]
Ni–Py–PDMS25000	0.04	2100	–	[72]
Co–Py–PDMS25000	0.05	1800	–	[73]
Ln–Py–PDMS	0.45	450	1.9	[20]
Ln–Bipy–PDMS	1.5	185	3.6	[74]
Ln–Tpy–PDMS (Mn = 5000, L–PU–Ln)	1.2	20	13.2	[75]
Ln–Tpy–PDMS (Mn = 25,000, H-PU–Ln)	0.58	498	1.7	[75]
PDMS-*block*-dithiothreitol	0.43	1500	–	[76]

wt*–weight percentage of PMMA.

## Data Availability

Not applicable.
